# Combining single-cell and transcriptomic analysis revealed the immunomodulatory effect of GOT2 on a glutamine-dependent manner in cutaneous melanoma

**DOI:** 10.3389/fphar.2023.1241454

**Published:** 2023-08-24

**Authors:** Lebin Song, Xiyi Wei, Xi Zhang, Yan Lu

**Affiliations:** ^1^ Department of Dermatology, The First Affiliated Hospital of Nanjing Medical University, Nanjing, China; ^2^ Department of Urology, The First Affiliated Hospital of Nanjing Medical University, Nanjing, China

**Keywords:** cutaneous melanoma, glutamine metabolism, prognostic model, GOT2, tumor immune, genetic mutation

## Abstract

**Background:** Reprogramming in glutamine metabolism is a hallmark of cancers, while its role in cutaneous melanoma has not been studied at great length.

**Methods:** Here, we constructed a glutamine metabolism-related prognostic signature in cutaneous melanoma with a variety of bioinformatics methods according to the glutamine metabolism regulatory molecules. Moreover, experimental verification was carried out for the key gene.

**Results:** We have identified two subgroups of cutaneous melanoma patients, each with different prognoses, immune characteristics, and genetic mutations. GOT2 was the most concerned key gene among the model genes. We verified its role in promoting tumor cell proliferation by CCK-8 and clone formation assays.

**Conclusion:** Our study cast new light on the prognosis of cutaneous melanoma, and the internal mechanism regulating glutamine metabolism of GOT2 may provide a new avenue for treating the cutaneous melanoma disease precisely.

## 1 Introduction

Melanoma is a potentially fatal malignancy resulting from the transformation and uncontrolled proliferation of pigment-producing melanocytes in the human epidermis (Curti and Faries, 2021). CM is the most aggressive form of melanoma due to its high incidence and metastasis rate. Only 14% of CM patients who develop metastasis survive beyond 5 years (Siegel et al., 2023). For advanced/metastatic CM, chemotherapy is usually ineffective, while radiotherapy is only effective in cases where brain metastases have occurred. A current guideline only recommends targeted therapy for patients with BRAF-mutated CM, whereas other types of mutations do not have sufficient evidence to support treatment recommendations (Seth et al., 2020). Recently, a series of important advances have been made in immunotherapy, such as anti-PD-1 and anti-CTLA-4 antibodies, which greatly reduce the mortality of CM (Seth et al., 2020; Huang and Zappasodi, 2022). Notably, due to specific environmental stresses induced by the treatment, many CM cases can develop genetic progression even under targeted or immunotherapies (Yen et al., 2021).

There is increasing evidence that during cancer development, not only do cancer cells themselves undergo metabolic alterations, but the TME also undergoes metabolic reprogramming due to metabolic competition (Pavlova and Thompson, 2016; Ruocco et al., 2019). Metabolic reprogramming of the TME would lead to invasive migration, metastasis, and even drug resistance of tumor cells, which is an important driver of cancer progression and immune impairment (Viale et al., 2014). For example, glycolysis is one of the key metabolic reprogramming processes in tumor cells, which provides immediate and sufficient energy for rapid proliferation, adaptation to hypoxic environments, and construction of an immunosuppressive TME. Lactate, a major metabolite of glycolysis involved in the construction of an acidic TME, promotes tumor progression and suppresses anti-tumor immunity by inhibiting T cells and PD-L1 upregulation (Feng et al., 2017). Therefore, metabolic reprogramming of tumor cells and immune cells is one of the key barriers to tumor immunotherapy (Kouidhi et al., 2018).

In hyperproliferative cells, glutamine plays an essential role in providing intermediates to the TCA cycle, which contributes to cell proliferation and biosynthesis (Yoo et al., 2020). As well as serving as a nitrogen donor for purines and pyrimidines, glutamine also serves as a precursor for GSH synthesis (Zhu et al., 2022). Glutamine is transported across membranes with the assistance of amino acid transporters and hydrolyzed into glutamate and ammonia through the catalysis of glutaminase. Then glutamate becomes α-KG catalyzed by dehydrogenase or aminotransferase and enters the TCA cycle (Yang et al., 2017). Meanwhile, glutamate can be oxidized into GSH via the glutamate-cysteine ligase, which participates in the immune defense, nutritional metabolism, and antioxidant function of cells by neutralizing mitochondrial ROS (Wang et al., 2019). Previous studies have reported that CM upregulates mitochondrial oxidative phosphorylation by enhancing glutamine anaplerotic metabolism, thereby promoting tumor cell resistance to targeted therapy (Haq et al., 2013; Hernandez-Davies et al., 2015; Baenke et al., 2016; Zhang et al., 2016; Vashisht Gopal et al., 2019). Several small molecule inhibitors have been utilized to take advantage of the “glutamine fragility” in cancer treatment due to the crucial role it plays in metabolic reprogramming (Li et al., 2019). Nonetheless, the clinical development of these drugs as monotherapies has been limited by toxicity or ineffectiveness (Wu et al., 2018; Leone et al., 2019).

It is important to note that glutamine is not just an important nutritional supplement for tumor cells, but also an essential component of immune cell function, including its role in activating T lymphocytes (Wang and Green, 2012; Cruzat et al., 2018). A recent study found that glutamine metabolizing enzyme inhibitors could promote Th1 and cytotoxic T-cell proliferation and viability by altering the epigenome (Johnson et al., 2018). The consumption and metabolism of glutamine may differ between tumor cells and TILs. Targeting the fragility of glutamine metabolism in tumor cells may change the fate of the TME and strengthen the anti-tumor ability of the TILs (Halama and Suhre, 2022; Ma et al., 2022).

In the current study, we established a glutamine metabolism risk score model based on GMRGs and evaluated the immune characteristics and tumor burden characteristics of the high- and low-GMRS groups under this characterization. The mechanism by which GOT2 regulates glutamine metabolism in tumor cells may provide new insights into targeted therapy for CM. Abbreviations and corresponding words and phrases used in this article can refer to Supplementary Table S1.

## 2 Materials and methods

### 2.1 Candidate data sources

The mRNA-seq datasets and clinical information of CM patients and normal controls were acquired from the TCGA-SKCM project and the GTEx website. The key enzymes involved in regulating glutamine metabolism were summarized from one previous study (Altman et al., 2016). We defined the genes encoding these 22 proteins as GMRGs. The single-cell count matrix was obtained from the GSE72056 (Tirosh et al., 2016), GSE115978 (Jerby-Arnon et al., 2018), and GSE120575 (Sade-Feldman et al., 2018) datasets from the GEO database. The gene expression matrix and clinical information used to validate the expression of the key gene and its correlation with clinicopathologic parameters were also obtained from the GSE3189 (Talantov et al., 2005), GSE15605 (Raskin et al., 2013), GSE114445 (Yan et al., 2019), and GSE46517 (Kabbarah et al., 2010) datasets from the GEO database. The online Xiantao tool (https://www.xiantao.love/products) is a comprehensive bioinformatics analysis website. Differential analysis, survival analysis, biofunctional enrichment analysis, and immune-related analyses of target genes were performed by this tool partly. The predicted 3D structure and immunohistochemical staining of the protein encoded by the key gene were obtained from the HPA database. The main R packages used in the analysis process were displayed in Supplementary Table S2.

### 2.2 Construction of the prognostic GMRG signature

The prognostic GMRGs correlated significantly with the OS of CM patients were screened out by univariate Cox analysis. The threshold of screening was *p* < 0.05. The composition of the optimal multi-gene signature was determined by successively applying the LASSO and multivariate Cox regression analyses (Gui and Li, 2005). The standardized expression value of each signature gene is multiplied by the corresponding regression coefficient and the results are summed to obtain the risk score of this signature, which is defined as GMRS. CM patients were stratified into two subgroups according to the median GMRSs. Survival analysis was performed to evaluate survival differences. The Time-dependent ROC curves were used to evaluate the predictive performance of our signature. The predictive independence of classical prognostic factors and our signature was identified by regression analyses.

### 2.3 Subgroup analysis of the gene signature

To confirm the consistency between our gene signature and conventional predictors, we performed a hierarchical difference analysis. Similarly, we also discussed the distribution of clinical parameters between GMRS subgroups. Moreover, the subgroup survival analysis was conducted based on different clinical parameters to verify the predictive ability of our model in different clinical signs.

Eight algorithms were used to compare the immune infiltration status between GMRS subgroups, including the ssGSEA algorithm and the corresponding gene sets used (Charoentong et al., 2017). The ESTIMATE algorithm allowed for a quantitative comparison of immune correlation status among GMRS subgroups using 4 kinds of scores (Yoshihara et al., 2013). The TIP tool was used to evaluate 23 standardized immune activity scores between GMRS subgroups during the cancer immune cycle (Xu et al., 2018).

### 2.4 Clustering analysis

The molecular subtypes of CM samples were identified by the consistent clustering method (Wilkerson and Hayes, 2010). The clustering algorithm is set to K-means and the coefficients *k* are set from 2 to 9. The optimal clustering result was presented as a heat map including the expression of modeled genes and the distribution of classical clinical parameters across cases. The distribution of different subtypes was explored by the t-SNE method (Laurens and Hinton, 2008).

### 2.5 Biological and mutational analysis

The Wilcoxon test with an adjusted *p* < 0.05 and a |log2 FC| > 1 threshold was used to screen out DEGs between clusters. The pathways enriched in each cluster were evaluated by GSVA. The GO and KEGG pathway enrichment analyses based on the above DEGs were also performed. The correlation between the expression of our key gene GOT2 and immune-related pathways from the KEGG database and Reactome database was evaluated by GSEA. The top 20 mutated genes were evaluated and the oncoplots were generated to provide a visual representation of the mutational landscape in GMRS subgroups. The survival analysis of the subgroups stratified by the TMB state of each sample was conducted.

### 2.6 Single-cell RNA-seq data processing

The raw data for single-cell analysis first underwent a process of normalization, dimensionality reduction, and clustering (Stuart et al., 2019). The criteria for cell filtering were more than 200 gene expressions and less than 20% mitochondrial gene expression. The criteria for gene filtering were expressed in more than 3 cells. The top 30 PCs for UMAP construction and a resolution of 0.4 for graph-based clustering were utilized to identify each cell cluster. The InferCNV method was utilized to estimate the CNV signal for individual cells (Patel et al., 2014). To define the reference cell-inferred copy number profiles, B cells, fibroblast, and T cells were utilized. Epithelial cells were used for the observations. According to the flow described by Sunny Z. Wu et al., (Wu et al., 2021), Epithelial cells were classified as either normal epithelial or malignant. The potential communication molecules between various cell subtypes were investigated using the CellPhone DB database (Vento-Tormo et al., 2018). Following that, we calculated the mean and the significance of cell communication and visualize the network plot according to the interaction matrix and the cell count matrix.

### 2.7 Cell culture and proliferation assay

Human melanoma cell lines A375 and SK-28 were purchased from the University of Colorado Cancer Center Cell Bank and cultured in DMEM medium supplemented with 10% FBS (Invitrogen, Carlsbad, CA, USA) at 37°C in a 5% CO_2_ atmosphere. siRNAs were used to knock down the expression level of GOT2 in cell lines. The siRNA sequences were as follows: siRNA#1-GCTACAAGGTTATCGGTATTA; siRNA#2-GCCTTCACTATGGTCTGCAAA. We used RT-qPCR to determine the knockdown efficiency of siRNA-GOT2. CCK8 and clone formation were applied to detect the proliferation activity of melanoma cells.

### 2.8 Statistical analysis

All analyses were performed using R software (version 4.2.2). All statistical tests were two-sided. *p*-value <0.05 or Spearman correlation coefficient >0.3 was considered statistically significant unless otherwise noted.

## 3 Results

### 3.1 Establishment of the GMRGs related prognostic signature

**FIGURE 1:**
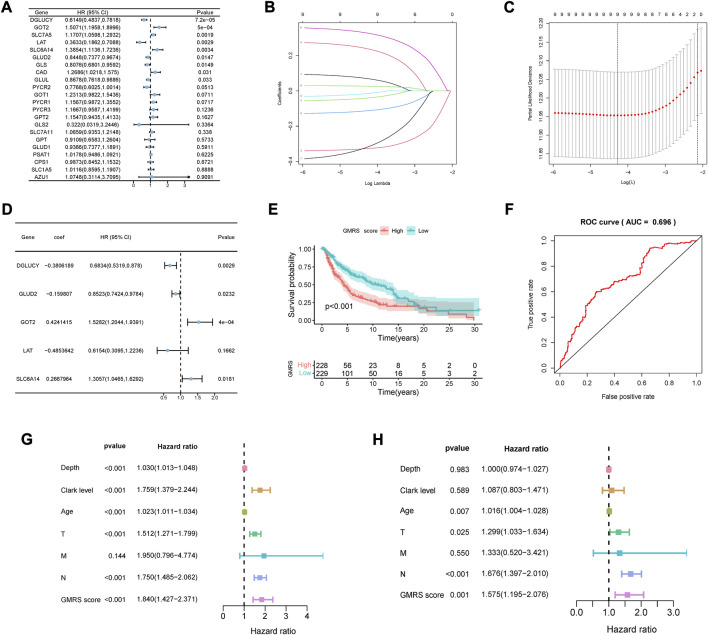
Development of the GMRS model. **(A)** Nine prognosis-related genes were selected by the univariate Cox regression analysis. **(B,C)** Nine prognosis-related genes underwent the LASSO regression analysis. **(D)** Five genes were selected with the best value of coefficient by the multivariate Cox regression analysis. **(E)** The K-M survival curve shows the poorer survival probability of CM patients in the high GMRS group (*p* < 0.001). **(F)** The AUC value of the ROC curve for predicting CM patients’ prognosis. **(G,H)** Predictive independence assessment of classical clinical predictors and our GMRS score by the **(G)** univariate and **(H)** multivariate Cox regression analysis.

The prognostic analysis was first carried out to filtrate GMRGs significantly related to outcomes in CM patients. As shown in the forest plot (Figure 1A), 9 GMRGs were found to be associated with prognosis (*p* < 0.05). 5 of the above 9 genes were regarded as protective genes and 6 were considered as risk genes. Then the multivariate Cox and LASSO regression analyses were applied in succession to single out the best model with the TCGA dataset (Figures 1B, C). Furthermore, A glutamine-metabolism-related prognostic signature for CM was constructed given the best coefficient value (Figure 1D). The risk score for our signature, which was defined as GMRS, was worked out through the established formula method:

$$GMRS=\left(-0.3806\right)*\exp^{DGLUCY}+\left(-0.1598\right)*\exp^{GLUD2}+\left(0.4241\right)*\exp^{GOT2}+\left(-0.4854\right)*\exp^{LAT}+\left(0.2668\right)*\exp^{SLC6A14}$$

The GMRS was then calculated for each case. Its mean threshold divided the CM patients into two subgroups (low-GMRS (n = 229) and high-GMRS (n = 228)). KM analysis confirmed a lower likelihood of survival in the high-GMRS group (*p* < 0.05, Figure 1E). An evaluation of the predictive performance was conducted with the time-dependent ROC curve of the model and in Figure 1F, the AUC reached 0.696. Univariate and multivariate Cox analyses were also performed to further explore the independent predictive ability of our signature. The univariate Cox regression analysis showed that Breslow depth, Clark level, age, pathologic T stage, pathologic N stage, and our GMRS were all significantly associated with survival (Figure 1G). However, when applying the corresponding multivariate Cox regression analysis, we found that only age (*p* = 0.007), pathologic T stage (*p* = 0.025), pathologic N stage (*P* < 0.001), and our GMRS (*p* = 0.001) were clinically independent (Figure 1H).

### 3.2 The relationship between GMRS and clinicopathologic factors

In order to evaluate the association between GMRS and clinicopathologic factors, we performed differential analysis and evaluated the distribution characteristics of classic clinicopathologic factors between two risk groups. The bar plots in Supplementary Figure S1A showed that patients with advanced T stage (*p* < 0.001), overall stage (*p* < 0.05), and Clark level significantly presented higher GMRSs. Consistent with this result, the high-GMRS group exhibited notably higher proportions of advanced T stage (*p* = 0.002), N stage (*p* = 0.043), overall stage (*p* = 0.004), and Clark level (*p* = 0.007) (Supplementary Figures S1A, B). Further, we performed a stratification analysis for survival probability to determine the prognostic power of our prognostic signature in subgroups of CM. As was shown in Supplementary Figure S1C, except for the subgroup of Clark level I/II (*p* = 0.088), patients in the high-GMRS group showed a more abysmal outcome than those in the low-GMRS group (all *p* < 0.05).

### 3.3 Mutation burden between high-GMRS and low-GMRS groups

**FIGURE 2:**
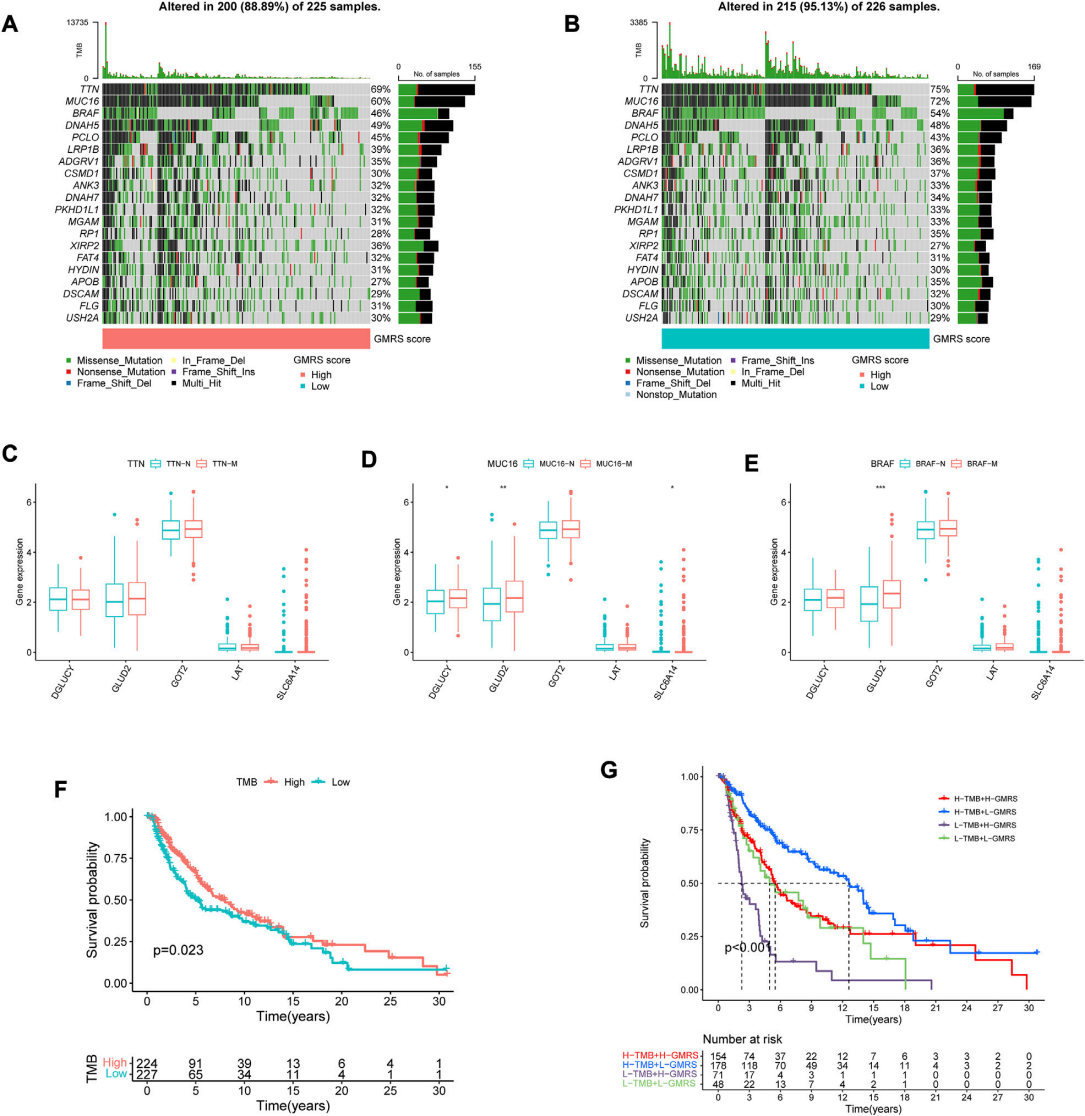
Interpretation of the gene mutation landscape between GMRS groups. **(A,B)** Oncoplots showing the top 20 mutation profiles in the **(A)** high-GMRS and **(B)** low-GMRS groups. The types of mutation in each gene and the corresponding percentage in the samples were also included. **(C–E)** The differential expression of the model genes among the subgroups stratified by the three top gene mutations **(C)** TTN; **(D)** MUC16; **(E)** BRAF). **(F)** The K-M survival curve shows the poorer survival probability of CM patients in the low TMB group. **(G)** The K-M survival curves show the survival differences between subgroups stratified by both TMB and GMRS scores in the CM cohort. * represents statistical *p-*value < 0.05, ** represents statistical *p-*value < 0.01, *** represents statistical *p-*value < 0.001.

Since a large variety of genetic alterations occur in the common progression trajectories of CM, the gene mutation status was comprehensively assayed in patients with different GMRSs (Shain and Bastian, 2016). The top 20 most mutative genes in each group were displayed (Figures 2A, B). Some of them overlapped, with a higher mutation frequency in the low-GMRS group. Figures 2C–E showed the differential expression of the model genes among the subgroups stratified by the three top gene mutations. Previous evidence revealed that the TMB severely affected the response to cancer immunotherapy and the long-term prognosis of tumor patients (Kang et al., 2020; Moldoveanu et al., 2022). According to our analysis, patients with high TMB experienced improved OS, relative to low TMB (*p* = 0.023, Figure 2F). Next, we evaluated the collaborative impact of our signature and TMB in predicting prognosis. The results showed that the high-GMRS group had worse OS than the low-GMRS group regardless of the TMB status, which implied that the prognostic value of our signature was not intervened by the TMB status of individuals (*p* < 0.001, Figure 2G).

### 3.4 Immune microenvironment difference between High-GMRS and Low-GMRS groups

**FIGURE 3:**
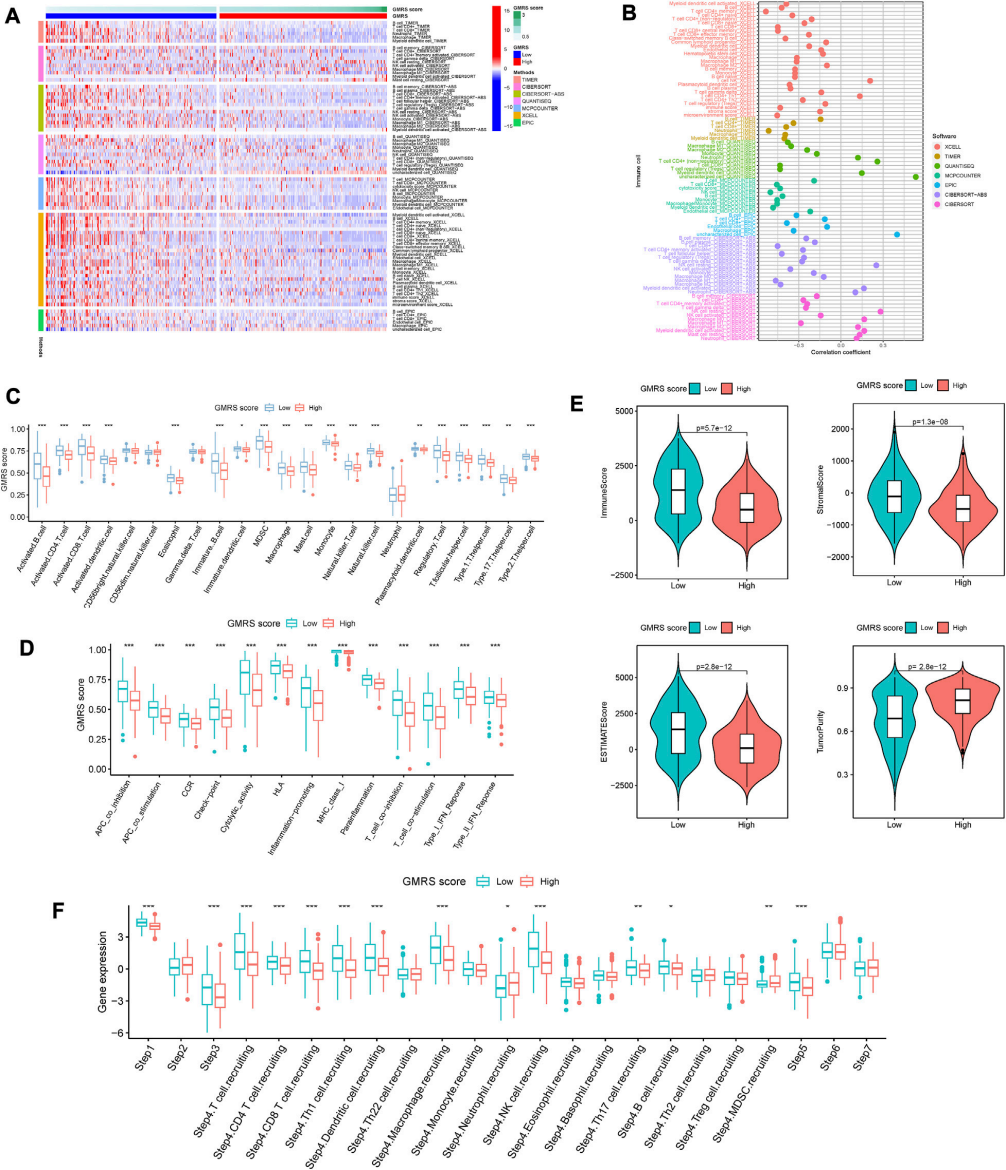
Comparison of the immune characteristics between risk groups. **(A)** Heatmap showing the distribution of tumor-infiltrating immune cells and GMRS scores by 7 mainstream algorithms. **(B)** Spearman correlation analysis of the tumor-infiltrating immune cells under GMRS score in CM. **(C)** Differential abundance analysis of tumor-infiltrating immune cells between GMRS groups using ssGSEA algorithm. **(D)** Differential immune function analysis between GMRS groups. **(E)** Differential analysis of immune-related scores between GMRS groups using the ESTIMATE algorithm. **(F)** Differential analysis of the anticancer immune functions of the cancer-immunity-cycle between GMRS groups. * represents statistical *p-*value < 0.05, ** represents statistical *p-*value < 0.01, *** represents statistical *p-*value < 0.001.

Considering CM as immune-related, and immunotherapy as preferable (Coit et al., 2013), we explored the difference in the immune infiltration situation between the two risk subgroups. The heatmap in Figure 3A showed visually some immune cells demonstrated significant differences according to most of the algorithms, such as B cells, CD8^+^ T cells, neutrophils, macrophages, and NK cells. The specific correlation analysis was shown in Figure 3B. And we can conclude that most immune cells have a significant negative correlation with the value of GMRS (r < −0.3).

Whereafter, the enrichments of 23 immune cell types and 13 immune-related functions were assessed between the low-GMRS and high-GMRS groups. The significant between-group difference showed up in most of the active immune cells and immunocompetent (*p* < 0.05), with a higher-level immune cell infiltration in the low-GMRS group (Figure 3C). Both groups demonstrated significant differences in all immune-related functions, such as checkpoint, inflammation-promoting, Type I IFN response, etc. (Figure 3D). By the ESTIMATE algorithm, we explored differences in immune-related scores between GMRS groups. Lower immune, stromal, and ESTIMATE scores were observed in the high-GMRS group, while its tumor purity scores were significantly higher (all *p* < 0.05, Figure 3E).

Chen et al. referred to seven sequential processes of antitumor immunity as the “cancer-immunity cycle” (Chen and Mellman, 2013). TIP (a web service for determining tumor immunophenotype profiling) was utilized to evaluate the anticancer immunological functions of the seven-step cancer-immunity cycle between GMRS groups (Xu et al., 2018). Our results in Figure 3F revealed that patients in low-GMRS groups owned significant functional enhancement in step 1 (release of cancer cell antigens), step 3 (priming and activation of effector T cell responses), step 4 (trafficking of immune cells to tumors), and step 5 (infiltration of immune cells into tumors). Specifically in step 4, the low-GMRS group presented the high activity of recruiting of 10 main immune cells, including T cells, macrophages, NK cells, Th17 cells, and so on. Only the process of MDSC recruiting was enhanced in high-GMRS groups. Taken together, the patients in the high-GMRS group were predisposed to an immune-silent microenvironment featuring full down-modulation of immune cell infiltration and immune functions, which may be followed by a dismal prognosis.

### 3.5 Identification of glutamine-metabolism-related molecular subtypes

According to the expression levels of five GMRGs from our prognostic signature, CM samples from TCGA were divided by clustering analysis. When *k* = 2, the consensus matrix heatmap in Supplementary Figure S2A exhibited high consistency between clusters. And the K-M survival curve in Supplementary Figure S2B showed a significant difference in survival between clusters (*p* = 0.015). The area under the CDF curve tended to be stable starting from k = 2 (Supplementary Figures S3A, B). The PCA plot in Supplementary Figure S2C also agreed with the clustering outcome of the two subtypes. The alluvial diagram demonstrated the distribution overlap of the CM samples between GMRS and molecular subtypes (Supplementary Figure S3C). The distribution of cluster A subtype samples was mainly comprised of high GMRSs, while cluster B subtype achieved a high ratio of the low-GMRS group in the samples. As shown in Supplementary Figure S2D, the heatmap illustrated the clinicopathologic features and expression patterns of five GMRGs.

To explore the between-group differences in biological features, GSVA was carried out to seek the enriched pathways in each cluster. Results with adjusted *p* < 0.05 were shown in Supplementary Figure S2E. Cluster A subtype was enriched in more tumor-related pathways such as “MYC_TARGETS_V2” (*adj.p* = 0.014), “P53_PATHWAY” (*adj.p* = 0.006), “WNT_BETA_CATENIN_SIGNALING” (*adj.p* = 0.031) and so on, which may account for the poor prognosis of patients in cluster A. More details can be obtained from Supplementary Table S3. Then, the enrichment analyses of the GO functions and KEGG pathways were performed. Enriched BPs of the DEGs mainly concluded “negative regulation of protein phosphorylation” and “cell-substrate adhesion”. Enriched of CCs were mainly in “cell-cell junction” and “collagen-containing extracellular matrix”. Enriched MFs consisted of “integrin binding” and “insulin-like growth factor I binding” (Supplementary Figure S2F). Enriched KEGG pathways were listed as “PI3K-Akt signaling pathway” and “Human papillomavirus infection” (Supplementary Figure S2G). These significantly enriched GO terms and KEGG pathways can offer a better understanding of the roles of these DEGs in the initiation and progression of CM.

### 3.6 Identification of the potential key gene in CM

**FIGURE 4:**
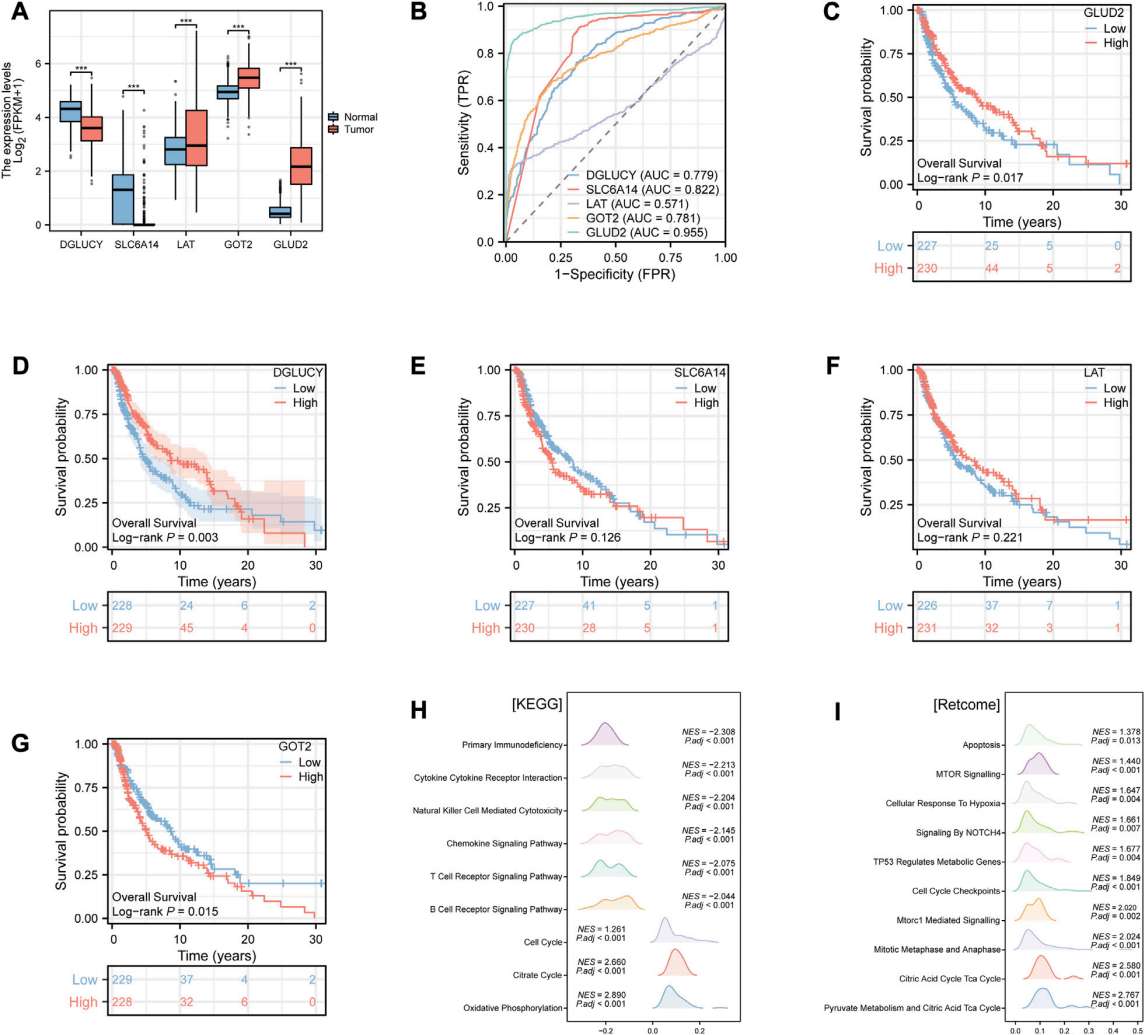
Identification of the potential key gene in CM by Xiantao tool. **(A)** Differential expression analysis of the model genes between CM and normal tissues. **(B)** ROC curves of diagnostic efficiency of the model genes. **(C–G)** K-M survival curves of the CM patients stratified by expression of model genes **(C)** GLUD2; **(D)** DGLUCY; **(E)** SLC6A14; **(F)** LAT; **(G)** GOT2), respectively. **(H–I)** Enrichment analysis of **(H)** KEGG and **(I)** Retcome pathways in CM patients according to the GOT2 expression. * represents statistical *p-*value < 0.05, ** represents statistical *p-*value < 0.01, *** represents statistical *p-*value < 0.001. NES, normalized enrichment score. P.adj, adjusted p value.

The differences in prognostic value and immune characteristics between the two clusters suggested that specific molecular biomarkers may contribute to CM patients’ prognostication. To find these key genes, we conducted the differential analysis and plotted the ROC curve of diagnostic efficiency. We found differential expressions of all five model genes between tumor and normal tissues. DGLUCY and SLC6A14 were lowly expressed, while those of LAT, GOT2, and GLUD2 were highly expressed in CM tissues (all *p* < 0.001, Figure 4A). The AUCs of DGLUCY, SLC6A14, GOT2, and GLUD2 were all greater than 0.7 (Figure 4B), implying that every one of them owned so adequate diagnostic efficiency to become an independent diagnostic biomarker. After a validation and survival analysis of these five genes in the TCGA cohort (Figures 4C–G), only GOT2 with a high expression was found to be associated with poor prognosis in CM patients (*p* = 0.015, Figure 4G). We also validated GOT2 expression in CM and its correlation with classical prognostic factors in external datasets. Among the GSE3189, GSE15605, and GSE114445 cohorts, the expression level of GOT2 was significantly higher in tumor tissues of CM than in adjacent normal skin tissues (all *p* < 0.05, Supplementary Figure S4A–C). Based on data from the GSE46517 cohort, we found that the expression of GOT2 was significantly higher in subgroups with more severe clinical predictors, including pathological T stage, distant metastasis, and overall stage (all *p* < 0.05, Supplementary Figure S4D–F).

Hence, we hypothesized that GOT2 might have a modest effect on tumor biological behavior in cancer cells. We then performed GSEA in CM to evaluate the gene enrichment according to GOT2 expression levels. In terms of KEGG pathways, overexpressed GOT2 was negatively associated with immune-related pathways (Figure 4H), such as “Primary Immunodeficiency” (*NES* = −2.308, *adj. p* < 0.001), “Cytokine Cytokine Receptor Interaction” (*NES* = −2.213, *adj. p* < 0.001), “Natural Killer Cell Mediated Cytotoxicity” (*NES* = −2.204, *adj. p* < 0.001). However, in terms of Retcome pathways, overexpressed GOT2 was positively associated with tumor-related pathways (Figure 4I), such as “MTOR Signaling” (*NES* = 1.44, *adj. p* < 0.001), “Cellular Response To Hypoxia” (*NES* = 1.647, *adj. p* = 0.004), “Signaling By NOTCH4” (*NES* = 1.661, *adj. p* = 0.007).

### 3.7 Potential function of GOT2 in regulating TME

**FIGURE 5:**
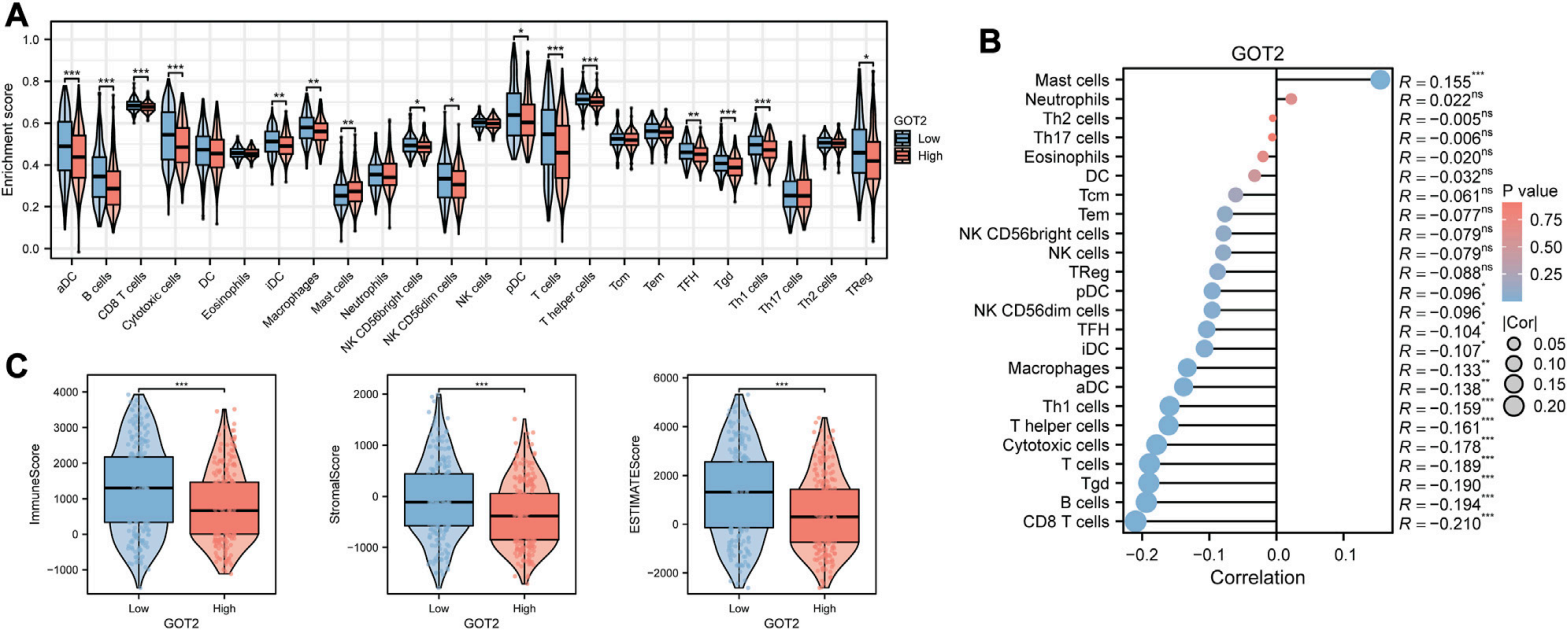
Comparison of the immune characteristics in GOT2 subgroups by Xiantao tool. **(A)** Differential abundance analysis of tumor-infiltrating immune cells between GOT2 groups using the ssGSEA algorithm. **(B)** Spearman correlation analysis of the tumor-infiltrating immune cells under GOT2 expression in CM. **(C)** Differential analysis of immune-related scores between GOT2 subgroups using the ESTIMATE algorithm. * represents statistical *p-*value < 0.05, ** represents statistical *p-*value < 0.01, *** represents statistical *p-*value < 0.001.

Since immune-related pathways were primarily enriched via GSEA, we decided to explore the regulatory effect of GOT2 on the TME. We first compared the differences in immune infiltration between high- and low-GOT2 expression groups. The infiltration levels of “NK CD56 bright cells”, “TFH”, “aDC”, “B cells”, “CD8 T cells”, “Cytotoxic cells”, “iDC”, “Macrophages”, “pDC”, “T helper cells”, “T cells”, “Tgd”, “Th1 cells”, and “Treg” were significantly lower in the high-GOT2 expression group (Figure 5A, all *p* < 0.05). The correlation between GOT2 and immune cells was also identified. The results in Figure 5B revealed that GOT2 was significantly negatively linked with the infiltration of most immune cells (|*R|*> 0.08, *p* < 0.05), such as CD8 T cells, B cells, Tgd, and T cells. Then, the ESTIMATE algorithm was conducted to calculate the immune and stromal scores. The high-GOT2 expression group showed lower immune, stromal, and ESTIMATE scores than the low-GOT2 expression group (all *p* < 0.001, Figure 5C). Our results demonstrated that GOT2 had the potential to become the diagnostic biomarker of CM and may pose as an immune-silent modulator in the TME of CM.

In addition, we also explored the ability of GOT2 as a predictor of immunotherapeutic response in CM. In three GEO cohorts that included immunotherapy-related information, we found that GOT2 expression did not appear to be significantly different between immunotherapy-responsive and non-responsive groups (all *p* > 0.05, Supplementary Figure S5A–C). Also, in the IMvigor210 cohort, there was no significant difference between the high- and low-GOT2 subgroups in the percentage of cases responding to immunotherapy (*p* > 0.05, Supplementary Figure S5D). Subsequently, we obtained the IPS scores of immunotherapy response to predict the efficacy of PD-1 and CTLA4 immune checkpoint blockers from the TCIA database. We found that only in the CTLA4-positive-PD-1-positive cohort, the IPS score was significantly higher in the low-GOT2 subgroup than in the high-GOT2 subgroup (Supplementary Figure S5E–H). The results suggested that the efficacy of immunotherapy could be enhanced by the combination of PD-1 inhibitor, CTLA4 inhibitor, and GOT2 inhibitor in CM.

### 3.8 Mapping GOT2 in single-cell data

**FIGURE 6:**
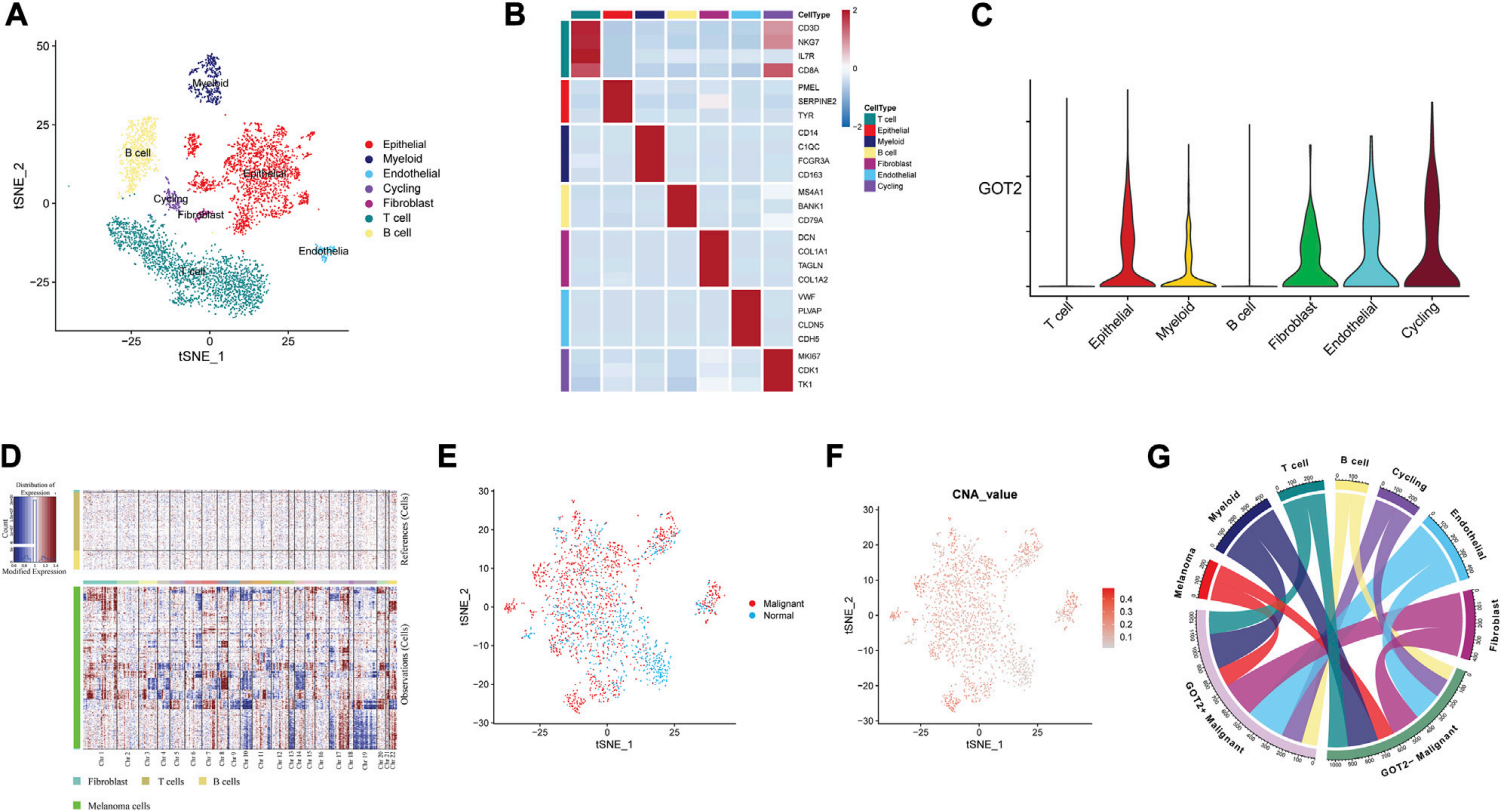
Mapping GOT2 in single-cell data. **(A)** A tSNE cluster view of 11701 cells was obtained from three GEO datasets. The identified clusters are annotated according to their origin. **(B)** The expression of marker genes for each cell type. **(C)** The density and distribution of GOT2 expression in different clusters. **(D)** Copy number analysis of the epithelial cells with the R package *inferCNV*. **(E,F)** The distribution of malignant cells and normal epithelial cells is based on the CNA values. **(G)** The multilineage interactome network among different cell clusters.

Because of the tumor heterogeneity, we sought to explore further at single-cell resolution. The scRNA-seq data of CM samples were obtained from three GEO datasets. Among the remaining cells after mass filtration, a total of 11701 single cells and 7 cell clusters were derived from tumors and non-malignant samples (Figure 6A). The fundamental markers of each major cell type were shown in the form of a heatmap in Figure 6B. The GOT2 expression was upregulated in several epithelial and stromal cells but downregulated in immune cells (Figure 6C). We further explored the genomic CNA in various cell subtypes to subdivide and identify malignant epithelial cells (Figure 6D). The tSNE plots in Figures 6E, F displayed the distribution of normal and malignant epithelial cells based on the CNA values. According to the expression abundance of GOT2, malignant epithelial cells were divided into GOT2+ and GOT2-malignant cells. Substantially high metabolic activity was observed in GOT2+ malignant cells compared with GOT2-malignant cells and normal cells.

### 3.9 Verification of GOT2 expression in CM

**FIGURE 7:**
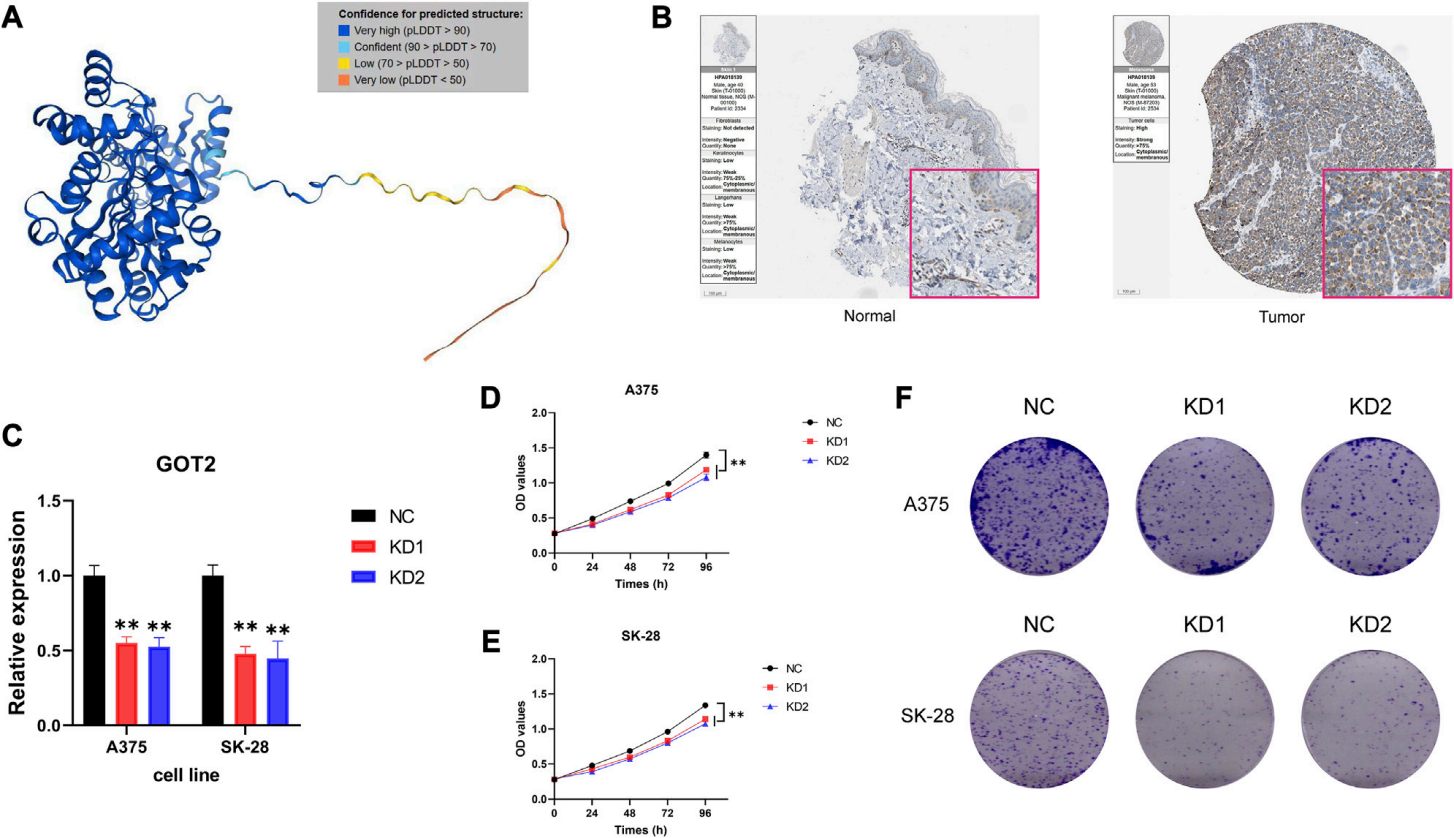
Verification of GOT2 expression in CM. **(A)** An accurately predicted complex structure of GOT2 protein by AlphaFold. **(B)** IHC staining of GOT2 in clinical CM tissues and normal skin tissues. **(C)** Verification of the knockdown efficiency of si-GOT2s by evaluating GOT2 mRNA expression using RT-qPCR. **(D–E)** Cell proliferation curve following transfection of si-GOT2 in **(D)** A375 and **(E)** SK-28 cells. **(F)** Comparison of the clone formation ability between negative control groups and GOT2 knockdown groups in A375 and SK-28 cells. NC, negative control; KD, knockdown; OD, optical density. * represents statistical *p-*value < 0.05, ** represents statistical *p-*value < 0.01, *** represents statistical *p-*value < 0.001.

The simulated visualization structure of GOT2 protein was predicted by AlphaFold and was displayed in Figure 7A (Tunyasuvunakool et al., 2021). The HPA database provided the immunohistochemical staining images of GOT2 protein. It was consistent that GOT2 was highly expressed in the CM tissues (Figure 7B). Subsequently, the RT-qPCR was performed and the si-GOT2 molecules were employed to effectively downregulate GOT2 mRNA expression in both A375 and SK-28 cell lines (all *p* < 0.01, Figure 7C). Furthermore, we used two methods to examine the effect of knocking down GOT2 on cell proliferation potential. Obviously, the cell proliferation was significantly restrained once A375 and SK-28 cells were transfected with si-GOT2 (all *p* < 0.01, Figures 7D, E). Additionally, the knockdown of GOT2 significantly inhibited the clone formation in A375 and SK-28 cells (Figure 7F). These results implied that GOT2 could promote the growth and proliferation of CM cells.

### 3.10 Pan-cancer analysis of GOT2 expression and prognostic significance

**FIGURE 8:**
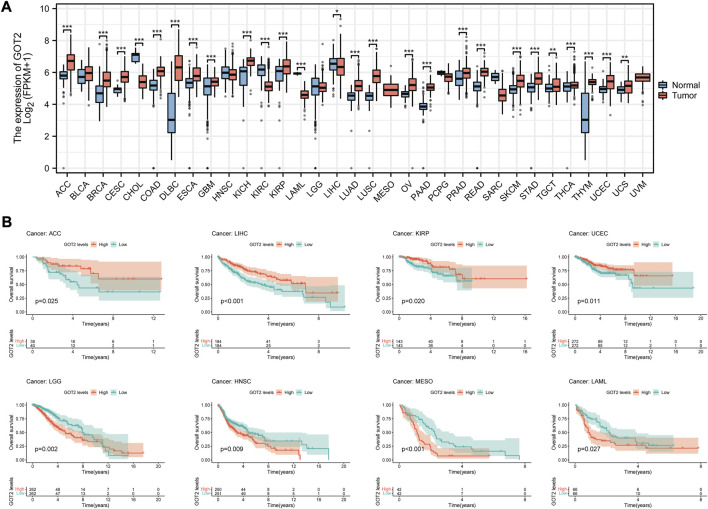
Pan-cancer analysis of GOT2 expression and prognostic significance. **(A)** Differential expression analysis of GOT2 between tumor tissues and corresponding adjacent normal tissues in 33 cancer types. **(B)** K-M survival curves of patients of 8 cancer types stratified by expression of GOT2. * represents statistical *p-*value < 0.05, ** represents statistical *p-*value < 0.01, *** represents statistical *p-*value < 0.001.

Based on TCGA data, we conducted the pan-cancer analysis to assess the expression and prognostic significance of GOT2 in various cancers. Differential expression analysis showed that GOT2 expression was significantly increased in 22 cancer types, such as ACC, BRCA, and CESC, compared with normal tissues (Figure 8A). In contrast, GOT2 expression was significantly decreased in 3 cancers, including CHOL, KIRC, and LIHC. Survival analyses showed that beyond CM, GOT2 expression also had prognostic significance in other 8 cancer types (all *p* < 0.05, Figure). GOT2 may be protective in ACC, LIHC, KIRP, and UCEC, while it is a risk factor for disease progression in LGG, HNSC, MESO, and LAML. All these data indicated that the dysregulation of this glutamic-oxaloacetic transaminase was common across several tumors, but the role of GOT2 in different tumors cannot be generalized, and further exploration was still needed.

## 4 Discussion

CM shares the oncogenic transformation features of glutamine addiction, like many other solid malignancies (Filipp et al., 2012). In other words, CM cells depend on glutamine for growth, irrespective of their oncogenic background. In a study quantitatively assessing glutamine metabolism in melanoma cell lines, it was shown that energy-producing anaplerosis and asparagine biosynthesis are responsible for CM cell growth (Ratnikov et al., 2015). The glutamine metabolism in CM cells is characterized by overexpression of genes involved in the production of proline from glutamate, thus increasing proline production by tumor cells in comparison with melanocytes (De Ingeniis et al., 2012). It is these metabolic changes that underlie the development of CM and that affect its response to treatment. Most tumors, in the meantime, trigger their own growth and development by remodeling the TME and recruiting corresponding cells, like TAMs and Tregs, which usually lead to poor outcomes (Watson et al., 2021; Shi et al., 2022). In CM, the TME consists of adjacent cells such as keratinocytes, adipocytes, immune cells, CAFs, and extracellular matrix elements (Mazurkiewicz et al., 2021). There is a strong correlation between immune cell infiltration in the TME and glutamine metabolism, according to several recent studies (Oh et al., 2020; Yang et al., 2021).

In our study, genes involved in glutamine metabolism in CM were based to generate a prognostic signature and evaluate its predictive value. The model possessed independent predictive power and was strongly correlated with other commonly used clinicopathological factors. In terms of immune function and infiltration, we found that patients in the high-GMRS group were developing an immune-silent microenvironment, which partly explained their poorer prognosis. By clustering CM cases according to the genes used for modeling, we defined two glutamine metabolism-related clusters. Among them, the CM patients in cluster A owned a high degree of overlap with the patients in the high-GMRS group, and both possessed poor prognoses. Mostly tumor-related signaling pathways were enriched in DEGs between the two clusters. Our findings further provided new insights into glutamine metabolism in melanoma.

Among these model genes, GOT2 stood out as an important enzyme for cellular redox homeostasis and aspartate production. As a crucial component of protein, purine, and pyrimidine nucleotide synthesis, aspartate also plays a crucial role in cell growth (Garcia-Bermudez et al., 2018). In our study, CM patients who expressed high levels of GOT2 had poor prognoses and low immune infiltration.

According to previous studies, GOT2 promoted the growth and proliferation of malignant tumors mainly through three pathways (Yang et al., 2015; Yang et al., 2018; Hong et al., 2019; Guan et al., 2021; Abrego et al., 2022). The first one is the supply of cellular building materials. Hong et al. reported that the protein BRCA1 and ZBRK1 can together form a complex that inhibits GOT2 transcription and translation. Due to the impairment of this complex, the production of GOT2 increased, which resulted in the rapid proliferation of breast cancer cells (Hong et al., 2019). The second one is the protection from oxidative damage. It is indispensable for cells to create NADPH and maintain the cellular redox state by glutamine carbon flow through GOT2. The knockdown of GOT2 in pancreatic ductal adenocarcinoma resulted in an increased production of ROS, which led to the cyclin-dependent kinase inhibitor p27-dependent senescence (Yang et al., 2018). The third one is the suppression of immune function. PPARδ is a lipid metabolism-related transcription factor. According to Abrego et al., GOT2 can shuttle to the nucleus and enhance PPARδ activity (Abrego et al., 2022). The translation products of many genes activated by PPARδ possess immunosuppressive properties, resulting in low immune infiltration as well as high tumor burden. We speculated that GOT2 could also play a cancer-promoting role through the above three pathways in CM.

However, GOT2 could exhibit a cancer-suppressive profile in some cancers. For instance, HCC cells exhibit downregulation of GOT2, and low GOT2 expression is associated with advanced disease progression (Li et al., 2022). Mechanistically, the reduction of GOT2 in HCC mediated the reprogramming of glutamine metabolism towards the synthesis of reduced GSH, which maintained redox homeostasis in tumor cells by resisting the ROS damage in HCC progression and stimulating the PI3K/AKT/mTOR signaling pathway thereby contributing to the malignant progression of HCC. The mechanisms behind GOT2 are still being explored, but it appears it is involved in reprogramming glutamine metabolism in order to promote cancer progression. This can be used as a therapeutic and diagnostic target for CM.

Admittedly, our research has some limitations. Our risk model included 5 genes, which may increase the cost of testing. We may be able to involve multiple clinicopathological parameters in the construction of the model to increase the practicality. The effect of GOT2 on immune function and immune cell infiltration may need more experiments to verify.

## 5 Conclusion

To summarize, we built up a brand-new prognostic model and stratified CM patients according to the model scores (GMRSs). Significant differences were found in prognosis, immune characteristics, and genomic mutation between these subgroups. Then, we identified the glutamine-metabolism-related molecular subtypes with different biological features. Furthermore, GMRGs like GOT2 could contribute to an in-depth understanding of the underlying mechanisms of CM and may become a new independent biomarker and target for the diagnosis and treatment of CM.

## Data Availability

The original contributions presented in the study are included in the article/Supplementary Material, further inquiries can be directed to the corresponding authors.

## References

[B1] Abrego J. Sanford-Crane H. Oon C. Xiao X. Betts C. B. Sun D. (2022). A cancer cell-intrinsic GOT2-pparδ Axis suppresses antitumor immunity. Cancer Discov. 12 (10), 2414–2433. 10.1158/2159-8290.Cd-22-0661 35894778 PMC9533011

[B2] Altman B. J. Stine Z. E. Dang C. V. (2016). From krebs to clinic: glutamine metabolism to cancer therapy. Nat. Rev. Cancer 16 (10), 619–634. 10.1038/nrc.2016.71 27492215 PMC5484415

[B3] Baenke F. Chaneton B. Smith M. Van Den Broek N. Hogan K. Tang H. (2016). Resistance to BRAF inhibitors induces glutamine dependency in melanoma cells. Mol. Oncol. 10 (1), 73–84. 10.1016/j.molonc.2015.08.003 26365896 PMC4717845

[B4] Charoentong P. Finotello F. Angelova M. Mayer C. Efremova M. Rieder D. (2017). Pan-cancer immunogenomic analyses reveal genotype-immunophenotype relationships and predictors of response to checkpoint blockade. Cell. Rep. 18 (1), 248–262. 10.1016/j.celrep.2016.12.019 28052254

[B5] Chen D. S. Mellman I. (2013). Oncology meets immunology: the cancer-immunity cycle. Immunity 39 (1), 1–10. 10.1016/j.immuni.2013.07.012 23890059

[B6] Coit D. G. Andtbacka R. Anker C. J. Bichakjian C. K. Carson W. E. Daud A. (2013). Melanoma, version 2.2013: featured updates to the NCCN guidelines. J. Natl. Compr. Canc Netw. 11 (4), 395–407. 10.6004/jnccn.2013.0055 23584343

[B7] Cruzat V. Macedo Rogero M. Noel Keane K. Curi R. Newsholme P. (2018). Glutamine: metabolism and immune function, supplementation and clinical translation. Nutrients 10 (11), 1564. 10.3390/nu10111564 30360490 PMC6266414

[B8] Curti B. D. Faries M. B. (2021). Recent advances in the treatment of melanoma. N. Engl. J. Med. 384 (23), 2229–2240. 10.1056/NEJMra2034861 34107182

[B9] De Ingeniis J. Ratnikov B. Richardson A. D. Scott D. A. Aza-Blanc P. De S. K. (2012). Functional specialization in proline biosynthesis of melanoma. PLoS One 7 (9), e45190. 10.1371/journal.pone.0045190 23024808 PMC3443215

[B10] Feng J. Yang H. Zhang Y. Wei H. Zhu Z. Zhu B. (2017). Tumor cell-derived lactate induces TAZ-dependent upregulation of PD-L1 through GPR81 in human lung cancer cells. Oncogene 36 (42), 5829–5839. 10.1038/onc.2017.188 28604752

[B11] Filipp F. V. Ratnikov B. De Ingeniis J. Smith J. W. Osterman A. L. Scott D. A. (2012). Glutamine-fueled mitochondrial metabolism is decoupled from glycolysis in melanoma. Pigment. Cell. Melanoma Res. 25 (6), 732–739. 10.1111/pcmr.12000 22846158 PMC3639292

[B12] Garcia-Bermudez J. Baudrier L. La K. Zhu X. G. Fidelin J. Sviderskiy V. O. (2018). Aspartate is a limiting metabolite for cancer cell proliferation under hypoxia and in tumours. Nat. Cell. Biol. 20 (7), 775–781. 10.1038/s41556-018-0118-z 29941933 PMC6030478

[B13] Guan H. Sun C. Gu Y. Li J. Ji J. Zhu Y. (2021). Circular RNA circ_0003028 contributes to tumorigenesis by regulating GOT2 via miR-1298-5p in non-small cell lung cancer. Bioengineered 12 (1), 2326–2340. 10.1080/21655979.2021.1935064 34077306 PMC8806680

[B14] Gui J. Li H. (2005). Penalized Cox regression analysis in the high-dimensional and low-sample size settings, with applications to microarray gene expression data. Bioinformatics 21 (13), 3001–3008. 10.1093/bioinformatics/bti422 15814556

[B15] Halama A. Suhre K. (2022). Advancing cancer treatment by targeting glutamine metabolism-A roadmap. Cancers (Basel) 14 (3), 553. 10.3390/cancers14030553 35158820 PMC8833671

[B16] Haq R. Shoag J. Andreu-Perez P. Yokoyama S. Edelman H. Rowe G. C. (2013). Oncogenic BRAF regulates oxidative metabolism via PGC1α and MITF. Cancer Cell. 23 (3), 302–315. 10.1016/j.ccr.2013.02.003 23477830 PMC3635826

[B17] Hernandez-Davies J. E. Tran T. Q. Reid M. A. Rosales K. R. Lowman X. H. Pan M. (2015). Vemurafenib resistance reprograms melanoma cells towards glutamine dependence. J. Transl. Med. 13, 210. 10.1186/s12967-015-0581-2 26139106 PMC4490757

[B18] Hong R. Zhang W. Xia X. Zhang K. Wang Y. Wu M. (2019). Preventing BRCA1/ZBRK1 repressor complex binding to the GOT2 promoter results in accelerated aspartate biosynthesis and promotion of cell proliferation. Mol. Oncol. 13 (4), 959–977. 10.1002/1878-0261.12466 30714292 PMC6441895

[B19] Huang A. C. Zappasodi R. (2022). A decade of checkpoint blockade immunotherapy in melanoma: understanding the molecular basis for immune sensitivity and resistance. Nat. Immunol. 23 (5), 660–670. 10.1038/s41590-022-01141-1 35241833 PMC9106900

[B20] Jerby-Arnon L. Shah P. Cuoco M. S. Rodman C. Su M. J. Melms J. C. (2018). A cancer cell program promotes T cell exclusion and resistance to checkpoint blockade. Cell. 175 (4), 984–997. 10.1016/j.cell.2018.09.006 30388455 PMC6410377

[B21] Johnson M. O. Wolf M. M. Madden M. Z. Andrejeva G. Sugiura A. Contreras D. C. (2018). Distinct regulation of Th17 and Th1 cell differentiation by glutaminase-dependent metabolism. Cell. 175 (7), 1780–1795. 10.1016/j.cell.2018.10.001 30392958 PMC6361668

[B22] Kabbarah O. Nogueira C. Feng B. Nazarian R. M. Bosenberg M. Wu M. (2010). Integrative genome comparison of primary and metastatic melanomas. PLoS One 5 (5), e10770. 10.1371/journal.pone.0010770 20520718 PMC2875381

[B23] Kang K. Xie F. Mao J. Bai Y. Wang X. (2020). Significance of tumor mutation burden in immune infiltration and prognosis in cutaneous melanoma. Front. Oncol. 10, 573141. 10.3389/fonc.2020.573141 33072607 PMC7531222

[B24] Kouidhi S. Ben Ayed F. Benammar Elgaaied A. (2018). Targeting tumor metabolism: A new challenge to improve immunotherapy. Front. Immunol. 9, 353. 10.3389/fimmu.2018.00353 29527212 PMC5829092

[B25] Laurens V. D. M. Hinton G. (2008). Visualizing Data using t-SNE. J. Mach. Learn. Res. 9 (2605), 2579–2605.

[B26] Leone R. D. Zhao L. Englert J. M. Sun I. M. Oh M. H. Sun I. H. (2019). Glutamine blockade induces divergent metabolic programs to overcome tumor immune evasion. Science 366 (6468), 1013–1021. 10.1126/science.aav2588 31699883 PMC7023461

[B27] Li L. Meng Y. Li Z. Dai W. Xu X. Bi X. (2019). Discovery and development of small molecule modulators targeting glutamine metabolism. Eur. J. Med. Chem. 163, 215–242. 10.1016/j.ejmech.2018.11.066 30522056

[B28] Li Y. Li B. Xu Y. Qian L. Xu T. Meng G. (2022). GOT2 silencing promotes reprogramming of glutamine metabolism and sensitizes hepatocellular carcinoma to glutaminase inhibitors. Cancer Res. 82 (18), 3223–3235. 10.1158/0008-5472.Can-22-0042 35895805

[B29] Ma G. Zhang Z. Li P. Zhang Z. Zeng M. Liang Z. (2022). Reprogramming of glutamine metabolism and its impact on immune response in the tumor microenvironment. Cell. Commun. Signal 20 (1), 114. 10.1186/s12964-022-00909-0 35897036 PMC9327201

[B30] Mazurkiewicz J. Simiczyjew A. Dratkiewicz E. Ziętek M. Matkowski R. Nowak D. (2021). Stromal cells present in the melanoma niche affect tumor invasiveness and its resistance to therapy. Int. J. Mol. Sci. 22 (2), 529. 10.3390/ijms22020529 33430277 PMC7825728

[B31] Moldoveanu D. Ramsay L. Lajoie M. Anderson-Trocme L. Lingrand M. Berry D. (2022). Spatially mapping the immune landscape of melanoma using imaging mass cytometry. Sci. Immunol. 7 (70), eabi5072. 10.1126/sciimmunol.abi5072 35363543

[B32] Oh M. H. Sun I. H. Zhao L. Leone R. D. Sun I. M. Xu W. (2020). Targeting glutamine metabolism enhances tumor-specific immunity by modulating suppressive myeloid cells. J. Clin. Invest. 130 (7), 3865–3884. 10.1172/jci131859 32324593 PMC7324212

[B33] Patel A. P. Tirosh I. Trombetta J. J. Shalek A. K. Gillespie S. M. Wakimoto H. (2014). Single-cell RNA-seq highlights intratumoral heterogeneity in primary glioblastoma. Science 344 (6190), 1396–1401. 10.1126/science.1254257 24925914 PMC4123637

[B34] Pavlova N. N. Thompson C. B. (2016). The emerging hallmarks of cancer metabolism. Cell. Metab. 23 (1), 27–47. 10.1016/j.cmet.2015.12.006 26771115 PMC4715268

[B35] Raskin L. Fullen D. R. Giordano T. J. Thomas D. G. Frohm M. L. Cha K. B. (2013). Transcriptome profiling identifies HMGA2 as a biomarker of melanoma progression and prognosis. J. Invest. Dermatol 133 (11), 2585–2592. 10.1038/jid.2013.197 23633021 PMC4267221

[B36] Ratnikov B. Aza-Blanc P. Ronai Z. A. Smith J. W. Osterman A. L. Scott D. A. (2015). Glutamate and asparagine cataplerosis underlie glutamine addiction in melanoma. Oncotarget 6 (10), 7379–7389. 10.18632/oncotarget.3132 25749035 PMC4480687

[B37] Ruocco M. R. Avagliano A. Granato G. Vigliar E. Masone S. Montagnani S. (2019). Metabolic flexibility in melanoma: A potential therapeutic target. Semin. Cancer Biol. 59, 187–207. 10.1016/j.semcancer.2019.07.016 31362075

[B38] Sade-Feldman M. Yizhak K. Bjorgaard S. L. Ray J. P. de Boer C. G. Jenkins R. W. (2018). Defining T cell states associated with response to checkpoint immunotherapy in melanoma. Cell. 175 (4), 998–1013. 10.1016/j.cell.2018.10.038 30388456 PMC6641984

[B39] Seth R. Messersmith H. Kaur V. Kirkwood J. M. Kudchadkar R. McQuade J. L. (2020). Systemic therapy for melanoma: ASCO guideline. J. Clin. Oncol. 38 (33), 3947–3970. 10.1200/jco.20.00198 32228358

[B40] Shain A. H. Bastian B. C. (2016). From melanocytes to melanomas. Nat. Rev. Cancer 16 (6), 345–358. 10.1038/nrc.2016.37 27125352

[B41] Shi Q. Shen Q. Liu Y. Shi Y. Huang W. Wang X. (2022). Increased glucose metabolism in TAMs fuels O-GlcNAcylation of lysosomal Cathepsin B to promote cancer metastasis and chemoresistance. Cancer Cell. 40 (10), 1207–1222.e10. 10.1016/j.ccell.2022.08.012 36084651

[B42] Siegel R. L. Miller K. D. Wagle N. S. Jemal A. (2023). Cancer statistics, 2023. CA Cancer J. Clin. 73 (1), 17–48. 10.3322/caac.21763 36633525

[B43] Stuart T. Butler A. Hoffman P. Hafemeister C. Papalexi E. Mauck W. M. 3rd (2019). Comprehensive integration of single-cell data. Cell. 177 (7), 1888–1902. 10.1016/j.cell.2019.05.031 31178118 PMC6687398

[B44] Talantov D. Mazumder A. Yu J. X. Briggs T. Jiang Y. Backus J. (2005). Novel genes associated with malignant melanoma but not benign melanocytic lesions. Clin. Cancer Res. 11 (20), 7234–7242. 10.1158/1078-0432.Ccr-05-0683 16243793

[B45] Tirosh I. Izar B. Prakadan S. M. Wadsworth M. H. 2nd Treacy D. Trombetta J. J. (2016). Dissecting the multicellular ecosystem of metastatic melanoma by single-cell RNA-seq. Science 352 (6282), 189–196. 10.1126/science.aad0501 27124452 PMC4944528

[B46] Tunyasuvunakool K. Adler J. Wu Z. Green T. Zielinski M. Žídek A. (2021). Highly accurate protein structure prediction for the human proteome. Nature 596 (7873), 590–596. 10.1038/s41586-021-03828-1 34293799 PMC8387240

[B47] Vashisht Gopal Y. N. Gammon S. Prasad R. Knighton B. Pisaneschi F. Roszik J. (2019). A novel mitochondrial inhibitor blocks MAPK pathway and overcomes MAPK inhibitor resistance in melanoma. Clin. Cancer Res. 25 (21), 6429–6442. 10.1158/1078-0432.Ccr-19-0836 31439581 PMC6825560

[B48] Vento-Tormo R. Efremova M. Botting R. A. Turco M. Y. Vento-Tormo M. Meyer K. B. (2018). Single-cell reconstruction of the early maternal-fetal interface in humans. Nature 563 (7731), 347–353. 10.1038/s41586-018-0698-6 30429548 PMC7612850

[B49] Viale A. Pettazzoni P. Lyssiotis C. A. Ying H. Sánchez N. Marchesini M. (2014). Oncogene ablation-resistant pancreatic cancer cells depend on mitochondrial function. Nature 514 (7524), 628–632. 10.1038/nature13611 25119024 PMC4376130

[B50] Wang R. Green D. R. (2012). Metabolic reprogramming and metabolic dependency in T cells. Immunol. Rev. 249 (1), 14–26. 10.1111/j.1600-065X.2012.01155.x 22889212 PMC3422760

[B51] Wang Y. Bai C. Ruan Y. Liu M. Chu Q. Qiu L. (2019). Coordinative metabolism of glutamine carbon and nitrogen in proliferating cancer cells under hypoxia. Nat. Commun. 10 (1), 201. 10.1038/s41467-018-08033-9 30643150 PMC6331631

[B52] Watson M. J. Vignali P. D. A. Mullett S. J. Overacre-Delgoffe A. E. Peralta R. M. Grebinoski S. (2021). Metabolic support of tumour-infiltrating regulatory T cells by lactic acid. Nature 591 (7851), 645–651. 10.1038/s41586-020-03045-2 33589820 PMC7990682

[B53] Wilkerson M. D. Hayes D. N. (2010). ConsensusClusterPlus: A class discovery tool with confidence assessments and item tracking. Bioinformatics 26 (12), 1572–1573. 10.1093/bioinformatics/btq170 20427518 PMC2881355

[B54] Wu C. Chen L. Jin S. Li H. (2018). Glutaminase inhibitors: A patent review. Expert Opin. Ther. Pat. 28 (11), 823–835. 10.1080/13543776.2018.1530759 30273516

[B55] Wu S. Z. Al-Eryani G. Roden D. L. Junankar S. Harvey K. Andersson A. (2021). A single-cell and spatially resolved atlas of human breast cancers. Nat. Genet. 53 (9), 1334–1347. 10.1038/s41588-021-00911-1 34493872 PMC9044823

[B56] Xu L. Deng C. Pang B. Zhang X. Liu W. Liao G. (2018). Tip: A web server for resolving tumor immunophenotype profiling. Cancer Res. 78 (23), 6575–6580. 10.1158/0008-5472.Can-18-0689 30154154

[B57] Yan B. Y. Garcet S. Gulati N. Kiecker F. Fuentes-Duculan J. Gilleaudeau P. (2019). Novel immune signatures associated with dysplastic naevi and primary cutaneous melanoma in human skin. Exp. Dermatol 28 (1), 35–44. 10.1111/exd.13805 30326165 PMC6333525

[B58] Yang H. Zhou L. Shi Q. Zhao Y. Lin H. Zhang M. (2015). SIRT3-dependent GOT2 acetylation status affects the malate-aspartate NADH shuttle activity and pancreatic tumor growth. Embo J. 34 (8), 1110–1125. 10.15252/embj.201591041 25755250 PMC4406655

[B59] Yang L. Venneti S. Nagrath D. (2017). Glutaminolysis: A hallmark of cancer metabolism. Annu. Rev. Biomed. Eng. 19, 163–194. 10.1146/annurev-bioeng-071516-044546 28301735

[B60] Yang S. Hwang S. Kim M. Seo S. B. Lee J. H. Jeong S. M. (2018). Mitochondrial glutamine metabolism via GOT2 supports pancreatic cancer growth through senescence inhibition. Cell. Death Dis. 9 (2), 55. 10.1038/s41419-017-0089-1 29352139 PMC5833441

[B61] Yang W. H. Qiu Y. Stamatatos O. Janowitz T. Lukey M. J. (2021). Enhancing the efficacy of glutamine metabolism inhibitors in cancer therapy. Trends Cancer 7 (8), 790–804. 10.1016/j.trecan.2021.04.003 34020912 PMC9064286

[B62] Yen I. Shanahan F. Lee J. Hong Y. S. Shin S. J. Moore A. R. (2021). ARAF mutations confer resistance to the RAF inhibitor belvarafenib in melanoma. Nature 594 (7863), 418–423. 10.1038/s41586-021-03515-1 33953400

[B63] Yoo H. C. Yu Y. C. Sung Y. Han J. M. (2020). Glutamine reliance in cell metabolism. Exp. Mol. Med. 52 (9), 1496–1516. 10.1038/s12276-020-00504-8 32943735 PMC8080614

[B64] Yoshihara K. Shahmoradgoli M. Martínez E. Vegesna R. Kim H. Torres-Garcia W. (2013). Inferring tumour purity and stromal and immune cell admixture from expression data. Nat. Commun. 4, 2612. 10.1038/ncomms3612 24113773 PMC3826632

[B65] Zhang G. Frederick D. T. Wu L. Wei Z. Krepler C. Srinivasan S. (2016). Targeting mitochondrial biogenesis to overcome drug resistance to MAPK inhibitors. J. Clin. Invest. 126 (5), 1834–1856. 10.1172/jci82661 27043285 PMC4855947

[B66] Zhu L. Zhu X. Wu Y. (2022). Effects of glucose metabolism, lipid metabolism, and glutamine metabolism on tumor microenvironment and clinical implications. Biomolecules 12 (4), 580. 10.3390/biom12040580 35454171 PMC9028125

