# Supplementary material for: Combining single-cell and transcriptomic analysis revealed the immunomodulatory effect of GOT2 on a glutamine-dependent manner in cutaneous melanoma
Source: Front Pharmacol. 2023 Aug 24;14:1241454. doi: 10.3389/fphar.2023.1241454 (PMC10483140; doi:10.3389/fphar.2023.1241454)
Supplement: Supplementary file 1 [file Table1.docx]

Table S1. Abbreviations and corresponding words and phrases used in this article.

| CM | cutaneous melanoma |
| --- | --- |
| PD-1 | programmed cell death protein 1 |
| CTLA-4 | cytotoxic T cell antigen 4 |
| TMB | tumor mutational burden |
| PD-L1 | programmed cell death 1 ligand 1 |
| TCA | tricarboxylic acid |
| GSH | glutathione |
| α-KG | alpha-ketoglutarate |
| ROS | reactive oxygen species |
| TILs | tumor-infiltrating lymphocytes |
| GMRG | glutamine metabolism-related gene |
| GMRS | risk score of glutamine metabolism-related signature |
| SKCM | Skin Cutaneous Melanoma |
| TCGA | The Cancer Genome Atlas Program |
| GTEx | the Genotype-Tissue Expression |
| GEO | Gene Expression Omnibus |
| HPA | Human Protein Atlas |
| OS | overall survival |
| LASSO | least absolute shrinkage and selection operator |
| ROC | receiver operating characteristic |
| AUC | area under the curve |
| ssGSEA | single-sample gene set enrichment analysis |
| ESTIMATE | Estimation of STromal and Immune cells in MAlignant Tumor tissues using Expression data |
| TIP | Tracking Tumor Immunotype |
| tSNE | t-distributed stochastic neighbour embedding |
| FC | fold charge |
| DEG | differentially expressed gene |
| GSVA | gene set variation analysis |
| GO | Gene Ontology |
| KEGG | Kyoto Encyclopedia of Genes and Genomes |
| GSEA | gene set enrichment analysis |
| PCs | principal components |
| UMAP | uniform manifold approximation and projection |
| CNV | copy number variation |
| DMEM | Dulbecco’s modified eagle medium |
| FBS | fetal bovine serum |
| siRNA | small interfering RNA |
| RT-qPCR | real-time quantitative polymerasechain reaction |
| CDF | cumulative distribution function |
| PCA | principal component analysis |
| BPs | biological processes |
| CCs | cellular components |
| MFs | molecular functions |
| Treg | regulatory T cell |
| scRNA | single-cell RNA |
| CNA | copy number alteration |
| ACC | Adrenocortical carcinoma |
| BRCA | Breast invasive carcinoma |
| CESC | Cervical squamous cell carcinoma and endocervical adenocarcinoma |
| CHOL | Cholangiocarcinoma |
| KIRC | Kidney renal clear cell carcinoma |
| LIHC | Liver hepatocellular carcinoma |
| KIRP | Kidney renal papillary cell carcinoma |
| UCEC | Uterine Corpus Endometrial Carcinoma |
| LGG | Brain Lower Grade Glioma |
| HNSC | Head and Neck squamous cell carcinoma |
| MESO | Mesothelioma |
| LAML | Acute Myeloid Leukemia |
| TAMs | tumor-associated macrophages |
| CAFs | cancer-associated fibroblasts |
| NADPH | nicotinamide adenine dinucleotide phosphate |
| PPARδ | peroxisome proliferator-activated receptor delta |
| HCC | hepatocellular carcinoma |
