# Supplementary figures and images for: Combining single-cell and transcriptomic analysis revealed the immunomodulatory effect of GOT2 on a glutamine-dependent manner in cutaneous melanoma
Source: Front Pharmacol. 2023 Aug 24;14:1241454. doi: 10.3389/fphar.2023.1241454 (PMC10483140; doi:10.3389/fphar.2023.1241454)

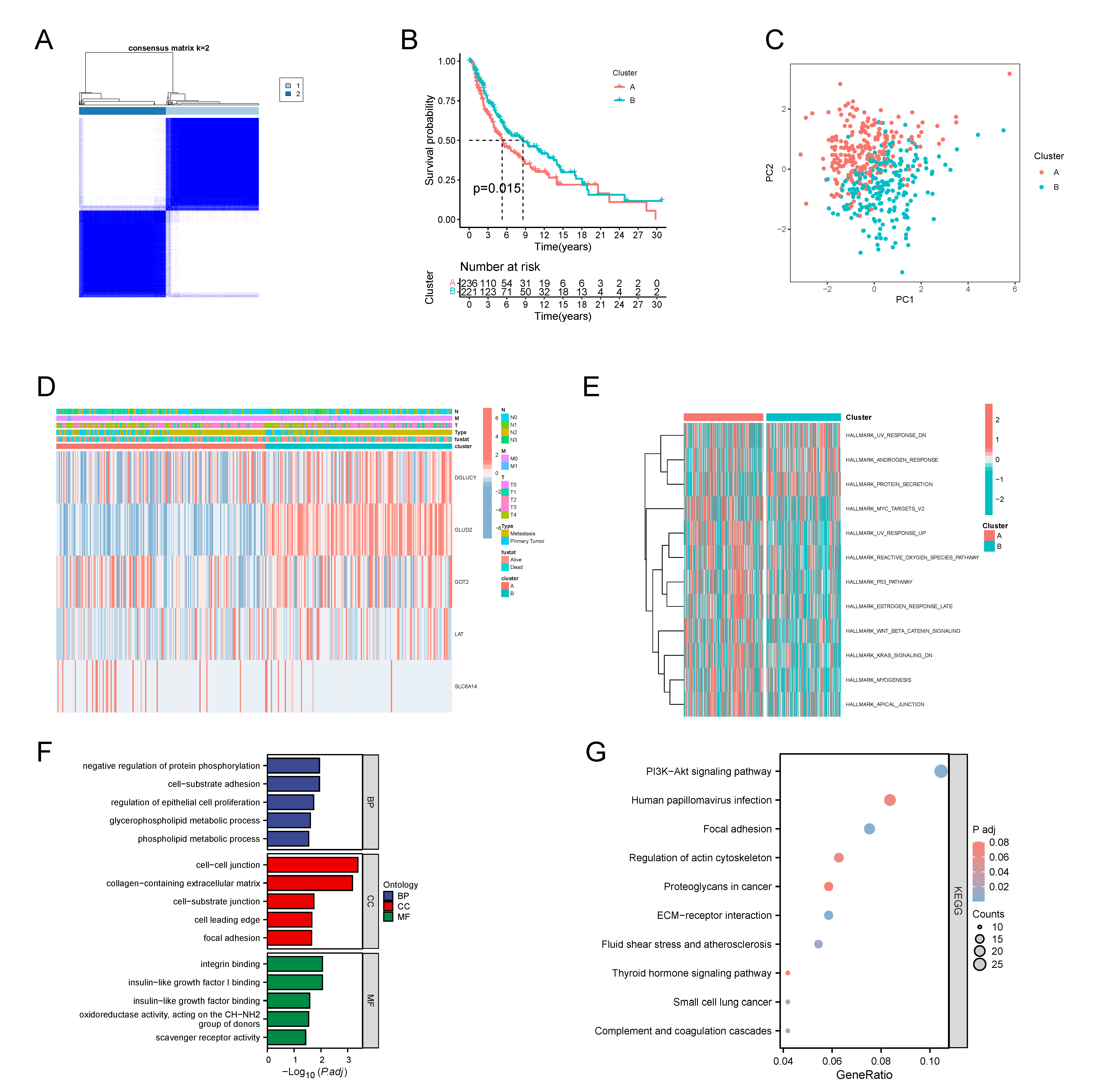

Supplement: Supplementary file 4 [file Image2.TIF]
