# Supplementary material for: Combining single-cell and transcriptomic analysis revealed the immunomodulatory effect of GOT2 on a glutamine-dependent manner in cutaneous melanoma
Source: Front Pharmacol. 2023 Aug 24;14:1241454. doi: 10.3389/fphar.2023.1241454 (PMC10483140; doi:10.3389/fphar.2023.1241454)
Supplement: Supplementary file 6 [file Table2.docx]

| **R package** | **Applied process** |
| --- | --- |
| *survival* | Survival Analysis |
| *survminer* | Survival Analysis |
| *glmnet* | LASSO Regression Analysis |
| *GSVA* | ssGSEA |
| *estimate* | ESTIMATE Analysis |
| *ConsensusClusterPlus* | Clustering Analysis |
| *pheatmap* | Heatmap Visualization |
| *t sne* | Dimensionality Reduction |
| *limma* | Differential Analysis |
| *ClusterProfiler* | GO and KEGG Enrichment Analysis |
| *maftools* | Mutational Analysis |
| *seurat* | Single-Cell Analysis |
| *InferCNV* | Single-Cell Analysis |
| *cellphonedb* | Single-Cell Analysis |
| *LRPlot* | Single-Cell Analysis |

Table S2. The main R packages used and the corresponding processes applied.
