# Supplementary material for: Combining single-cell and transcriptomic analysis revealed the immunomodulatory effect of GOT2 on a glutamine-dependent manner in cutaneous melanoma
Source: Front Pharmacol. 2023 Aug 24;14:1241454. doi: 10.3389/fphar.2023.1241454 (PMC10483140; doi:10.3389/fphar.2023.1241454)
Supplement: Supplementary file 7 [file Table3.docx]

Table S3. Signaling pathways with significant differences between the two glutamine-metabolism-related molecular subtypes

| ID | logFC | AveExpr | t | P.Value | adj.P.Val | B |
| --- | --- | --- | --- | --- | --- | --- |
| HALLMARK_ANDROGEN_RESPONSE | 0.108422 | -0.04007 | 5.198131 | 3.03E-07 | 1.05E-05 | 6.294426 |
| HALLMARK_KRAS_SIGNALING_DN | -0.08808 | -0.03036 | -5.13009 | 4.27E-07 | 1.05E-05 | 5.967429 |
| HALLMARK_PROTEIN_SECRETION | 0.137158 | -0.03366 | 5.009588 | 7.77E-07 | 1.27E-05 | 5.39769 |
| HALLMARK_UV_RESPONSE_UP | -0.06791 | -0.05068 | -3.85202 | 0.000134 | 0.001637 | 0.556644 |
| HALLMARK_MYOGENESIS | -0.07449 | -0.04244 | -3.63895 | 0.000305 | 0.002985 | -0.20612 |
| HALLMARK_UV_RESPONSE_DN | 0.081506 | -0.04375 | 3.549705 | 0.000425 | 0.003472 | -0.51344 |
| HALLMARK_REACTIVE_OXYGEN_SPECIES_PATHWAY | -0.09306 | -0.05325 | -3.49492 | 0.00052 | 0.003639 | -0.69852 |
| HALLMARK_P53_PATHWAY | -0.05796 | -0.04602 | -3.27239 | 0.001146 | 0.006604 | -1.42216 |
| HALLMARK_APICAL_JUNCTION | -0.06729 | -0.04485 | -3.25598 | 0.001213 | 0.006604 | -1.47372 |
| HALLMARK_MYC_TARGETS_V2 | -0.10783 | -0.03554 | -3.00083 | 0.002838 | 0.013905 | -2.2436 |
| HALLMARK_WNT_BETA_CATENIN_SIGNALING | -0.05728 | -0.05137 | -2.68989 | 0.007406 | 0.031379 | -3.09999 |
| HALLMARK_ESTROGEN_RESPONSE_LATE | -0.05195 | -0.04436 | -2.67734 | 0.007685 | 0.031379 | -3.13268 |
